# Retraction: Corrigendum: *Staphylococcus parequorum* sp. nov. and *Staphylococcus halotolerans* sp. nov., isolated from traditional Korean soybean foods

**DOI:** 10.71150/jm.2510100

**Published:** 2025-10-22

**Authors:** Ju Hye Baek, Dong Min Han, Dae Gyu Choi, Chae Yeong Moon, Jae Kyeong Lee, Chul-Hong Kim, Jung-Woong Kim, Che Ok Jeon

**Affiliations:** Department of Life Science, Chung-Ang University, Seoul 06974, Republic of Korea

**Retraction: Journal of Microbiogy. 2025. 63: e2509100.**

https://doi.org/10.71150/jm.2509100

­

The above corrigendum has been retracted from *Journal of Microbiology* at the request of the corresponding author. After publication, it was found that the corrigendum did not include the entire protologue, and thus the content was incomplete.

All of the authors agreed to this retraction. The authors regret any inconvenience that this may cause and sincerely apologize to the readers, reviewers, and editors of *Journal of Microbiology*.

A corrected corrigendum including the complete protologue will be published separately.

